# Supplementary material for: Serum GFAP and NfL augment a metabolomics-driven strategy for long-term prediction of multiple sclerosis progression
Source: Commun Med (Lond). 2026 Feb 25;6:182. doi: 10.1038/s43856-026-01453-5 (PMC13046805; doi:10.1038/s43856-026-01453-5)
Supplement: Supplementary file 2 — Supplementary Information [file 43856_2026_1453_MOESM2_ESM.pdf]

Supporting Information

# Title: Serum GFAP and NfL Augment a Metabolomics-Driven Strategy for Long-Term Prediction of Multiple Sclerosis Progression

***Authors:* *Tereza Kacerova*[1], *Eline Willemse*[2,3,4,5], *Johanna Oechtering*[2,3,4], *Daniel E. Radford-Smith*[6], *Wenzheng Xiong*[6], *Megan Sealey*[6], *Luisa Saldana*[7], *Aleksandra Maleska Maceski*[2,3,4], *Tianrong Yeo*[8,9,10], *Gabriele DeLuca*[7], *Jacqueline Palace*[7], *David Leppert*[2,3,4], *Jens Kuhle*[2,3,4], *Daniel C. Anthony*[6*], *Fay Probert*[1]**

***Addresses***

[1] Chemistry Research Laboratory, Department of Chemistry, University of Oxford, Oxford OX1 3TA, UK
[2] Department of Neurology, University Hospital and University of Basel, Basel, Switzerland
[3] Multiple Sclerosis Centre, Departments of Biomedicine and Clinical Research, University Hospital and University of Basel, Basel, Switzerland
[4] Research Center for Clinical Neuroimmunology and Neuroscience Basel, University Hospital and University of Basel, Switzerland
[5] Department of Clinical Research, University Hospital Basel, University of Basel, Basel, Switzerland
[6] Department of Pharmacology, University of Oxford, Oxford OX1 3QT, UK
[7] Nuffield Department of Clinical Neurosciences, John Radcliffe Hospital, University of Oxford, Oxford OX3 9DU, UK.
[8] Department of Neurology, National Neuroscience Institute, 11 Jalan Tan Tock Seng, Singapore 308433, Singapore
[9] Duke-NUS Medical School, 8 College Road, Singapore 169857, Singapore
[10] Lee Kong Chian School of Medicine (Nanyang Technological University), 11 Mandalay Road, Singapore 308232, Singapore

***Corresponding authors***

***Daniel C. Anthony** daniel.anthony@pharm.ox.ac.uk
Tel: +44(0)1865 281135

## *Metabolomics discriminates between RRMS and SPMS*

**Supplementary Table 1** The demographic characteristics (all results are presented as mean ± standard error of the mean (SEM)) of stable RRMS and SPMS individuals involved in the initial multivariate model. In this analysis, all stable RRMS and stable SPMS individuals were incorporated (only individuals transitioning from RRMS to SPMS were deliberately excluded from the analysis).

| Parameter | RRMS | SPMS |
|---|---|---|
| *N* | 20 | 9 |
| Female (%) | 13 (65.0%) | 3 (33.3%) |
| EDSS first sample | 3.3 ± 1.1 | 4.1 ± 1.1 |
| EDSS last sample | 3.0 ± 1.6 | 6.4 ± 0.9 |
| Age first sample | 44.1 ± 9.4 | 50.9 ± 13.9 |
| Age last sample | 50.6 ± 9.6 | 57.8 ± 14.8 |
| Treatment – baseline | Orals (6)<br>Monoclonal antibodies (7)<br>Platform (4)<br>Untreated (3) | Orals (1)<br>Monoclonal antibodies (1)<br>Platform (1)<br>Untreated (6) |

**OPLS-DA Model parameters:**

Model accuracy: 68.7 ± 2.4%

Model sensitivity: 67.9 ± 3.8%

Model specificity: 70.6 ± 3.3%

**Random Forest Model parameters:**

Model accuracy: 68.9 ± 2.7%

Model sensitivity: 69.8 ± 3.9%

Model specificity: 69.2 ± 3.8%

### *Metabolomics discriminates between RRMS and SPMS*

**Supplementary Table 2** Demographic characteristics (all results are presented as mean ± SEM) of stable RRMS and SPMS individuals used for the RRMS vs SPMS multivariate model (a size-matched *sub*-cohort was selected)

| Parameter | RRMS | SPMS |
|---|---|---|
| *N* | 10 | 9 |
| Female (%) | 7 (70.0%) | 3 (33.3%) |
| EDSS first sample | 2.8 ± 0.7 | 4.1 ± 1.1 |
| EDSS last sample | 2.1 ± 1.1 | 6.4 ± 0.9 |
| Age first sample | 42.4 ± 8.6 | 50.9 ± 13.9 |
| Age last sample | 48.6 ± 8.7 | 57.8 ± 14.8 |
| Treatment | Orals (3)<br>Monoclonal antibodies (2)<br>Platform (3)<br>Untreated (2) | Orals (1)<br>Monoclonal antibodies (1)<br>Platform (1)<br>Untreated (6) |

OPLS-DA model discriminating between "pure" RRMS and SPMS individuals was constructed. The *sub*-cohort comprises 10 participants with stable RRMS (selected based on the baseline EDSS score) and 9 participants with SPMS. Note: multiple samples from each individual (collected at different time points over an 8-year period) were used to construct this multivariate model to ensure adequate statistical power.

**OPLS-DA Model parameters:**

Model accuracy: 74.6 ± 2.3%

Model sensitivity: 73.9 ± 3.1%

Model specificity: 77.1 ± 6.2%

AUC (multivariable ROC – metabolites): 0.886 (95% CI 0.834 – 0.957)

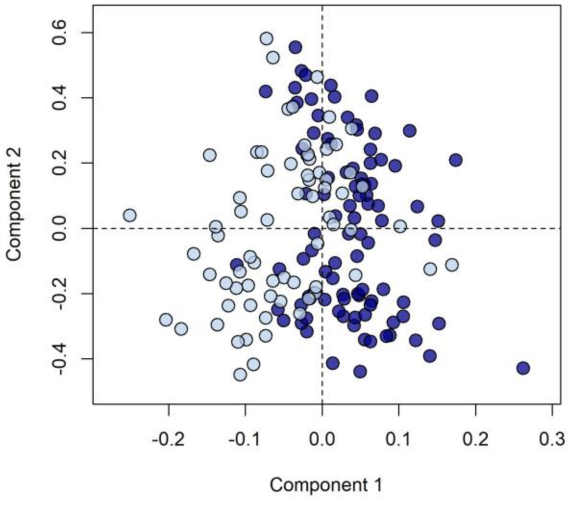


**Supplementary Fig. 1** OPLS-DA model discriminating between RRMS (dark blue) and SPMS (light blue) individuals

**Supplementary Table 3** List of the twenty most discriminatory compounds for the RRMS vs SPMS model based on the OPLS-DA algorithm (the fold change is given for SPMS relative to RRMS (SPMS/RRMS))

| Metabolite | NMR Bin | VIP Rank | VIP Score | Fold change |
|---|---|---|---|---|
| Mobile (–$CH_2$–)n (LDL dominated) | [1.28..1.30] | 1 | 2.889 | 0.921 |
| Mobile (–$CH_2$–)n (LDL dominated) | [1.30..1.32] | 2 | 2.418 | 0.954 |
| Mobile (–$CH_2$–)n (LDL dominated) | [1.26..1.28] | 3 | 2.235 | 0.925 |
| Valine | [0.98..1.00] | 4 | 1.812 | 1.104 |
| Glutamine | [2.12..2.14] | 5 | 1.810 | 1.076 |
| Glucose | [3.76..3.78] | 6 | 1.762 | 1.048 |
| Glutamine | [2.48..2.50] | 7 | 1.683 | 1.128 |
| Alanine | [1.48..1.50] | 8 | 1.654 | 1.036 |
| Mobile (–$CH_3$)n (HDL dominated) | [0.88..0.90] | 9 | 1.621 | 0.936 |
| GlycA | [3.66..3.68] | 10 | 1.601 | 1.069 |
| Glucose | [3.88..3.90] | 11 | 1.589 | 1.046 |
| GlycA/=CH–C**$H_2$**–$CH_2$– | [2.04..2.06] | 12 | 1.543 | 1.024 |
| Mobile (–$CH_3$)n (HDL dominated) | [0.86..0.88] | 13 | 1.411 | 1.023 |
| Valine | [1.02..1.04] | 14 | 1.399 | 1.064 |
| GlycA | [2.00..2.02] | 15 | 1.345 | 0.963 |
| Glutamine | [2.44..2.46] | 16 | 1.339 | 1.123 |
| Glucose | [3.70..3.72] | 17 | 1.282 | 1.036 |
| Glucose | [3.90..3.92] | 18 | 1.235 | 1.032 |
| Alanine | [1.46..1.48] | 19 | 1.176 | 1.052 |
| Glutamine | [2.46..2.48] | 20 | 1.152 | 1.113 |

(*N.B.* the LDLs and HDLs regions were assigned based on previous signal deconvolution studies (Yeo *et al.* (2021) in *Brain Commun* 3, DOI: 10.1093/braincomms/fcab240).

**Random Forest Model parameters:**

Model accuracy: 74.9 ± 2.6%

Model sensitivity: 74.4 ± 4.0%

Model specificity: 76.7 ± 3.4%

**Supplementary Table 4** List of the twenty most discriminatory compounds for the RRMS vs SPMS model based on the Random Forest algorithm

| Metabolite | NMR Bin | VIP Rank | VIP Score |
|---|---|---|---|
| Mobile (–$CH_3$)n (HDL dominated) | [0.82....0.84] | 1 | 3.221 |
| Mobile (–$CH_3$)n (HDL dominated) | [0.84....0.86] | 2 | 1.566 |
| Mobile (–$CH_3$)n (HDL dominated) | [0.88....0.90] | 3 | 1.423 |
| Valine | [0.98....1.00] | 4 | 1.367 |
| Mobile (–$CH_3$)n (VLDL dominated) | [0.90....0.92] | 5 | 1.366 |
| Mobile (–$CH_3$)n (HDL dominated) | [0.86....0.88] | 6 | 1.261 |
| Valine | [1.04....1.06] | 7 | 1.259 |
| Valine | [1.02....1.04] | 8 | 1.123 |
| Mobile (–$CH_2$–)n (LDL dominated) | [1.22....1.24] | 9 | 0.998 |
| Mobile (–$CH_2$–)n (LDL dominated) | [1.24....1.26] | 10 | 0.885 |
| Mobile (–$CH_2$–)n (LDL dominated) | [1.26....1.28] | 11 | 0.807 |
| Mobile (–$CH_2$–)n (LDL dominated) | [1.28....1.30] | 12 | 0.737 |
| Mobile (–$CH_2$–)n (LDL dominated)/lactate | [1.30....1.32] | 13 | 0.685 |
| Alanine | [1.46....1.48] | 14 | 0.641 |
| Lactate | [4.12....4.14] | 15 | 0.608 |
| Glucose | [3.78....3.80] | 16 | 0.607 |
| Glucose | [3.76....3.78] | 17 | 0.606 |

***Metabolomics discriminates between RRMS and SPMS – Demographics of the eight individuals changing MS stage during the follow-up period***

**Supplementary Table 5** Demographic characteristics of individuals transitioning from RRMS to SPMS (the patient numbers correspond to the numbering in Fig. 1)

| Patient | Class | Sex | Age sample 1 | Age last sample | EDSS sample 1 | EDSS last sample | Samples RRMS | Samples SPMS |
|---|---|---|---|---|---|---|---|---|
| 1 | Progressor | F | 43.8 | 51.3 | 2.5 | 6.0 | 5 | 10 |
| 2 | Progressor | F | 47.5 | 55 | 6.0 | 8.0 | 4 | 9 |
| 3 | Progressor | F | 43.9 | 49.1 | 3.0 | 4.5 | 4 | 7 |
| 4 | Progressor | F | 40.8 | 48.9 | 2.0 | 4.5 | 1 | 15 |
| 5 | Progressor | F | 58.9 | 64.3 | 3.5 | 6.0 | 2 | 8 |
| 6 | Progressor | F | 41.7 | 44.5 | 3.5 | 6.5 | 3 | 3 |
| 7 | Progressor | F | 42.3 | 50.7 | 4.0 | 6.5 | 7 | 3 |
| 8 | Progressor | M | 40.6 | 47.7 | 6.5 | 8.0 | 1 | 9 |
| Average | NA | NA | **44.9** | **51.4** | **3.9** | **6.3** | | |
| St. dev. | NA | NA | **6.1** | **6.0** | **1.6** | **1.5** | | |

## *Metabolomics facilitates the staging of multiple sclerosis in an individual*

Individuals diagnosed with RRMS who remained stable (classified as RRMS throughout the follow-up period) exhibited a metabolomic signature with minimal fluctuations compared to those who transitioned from RRMS to SPMS (refer to Fig. 1).

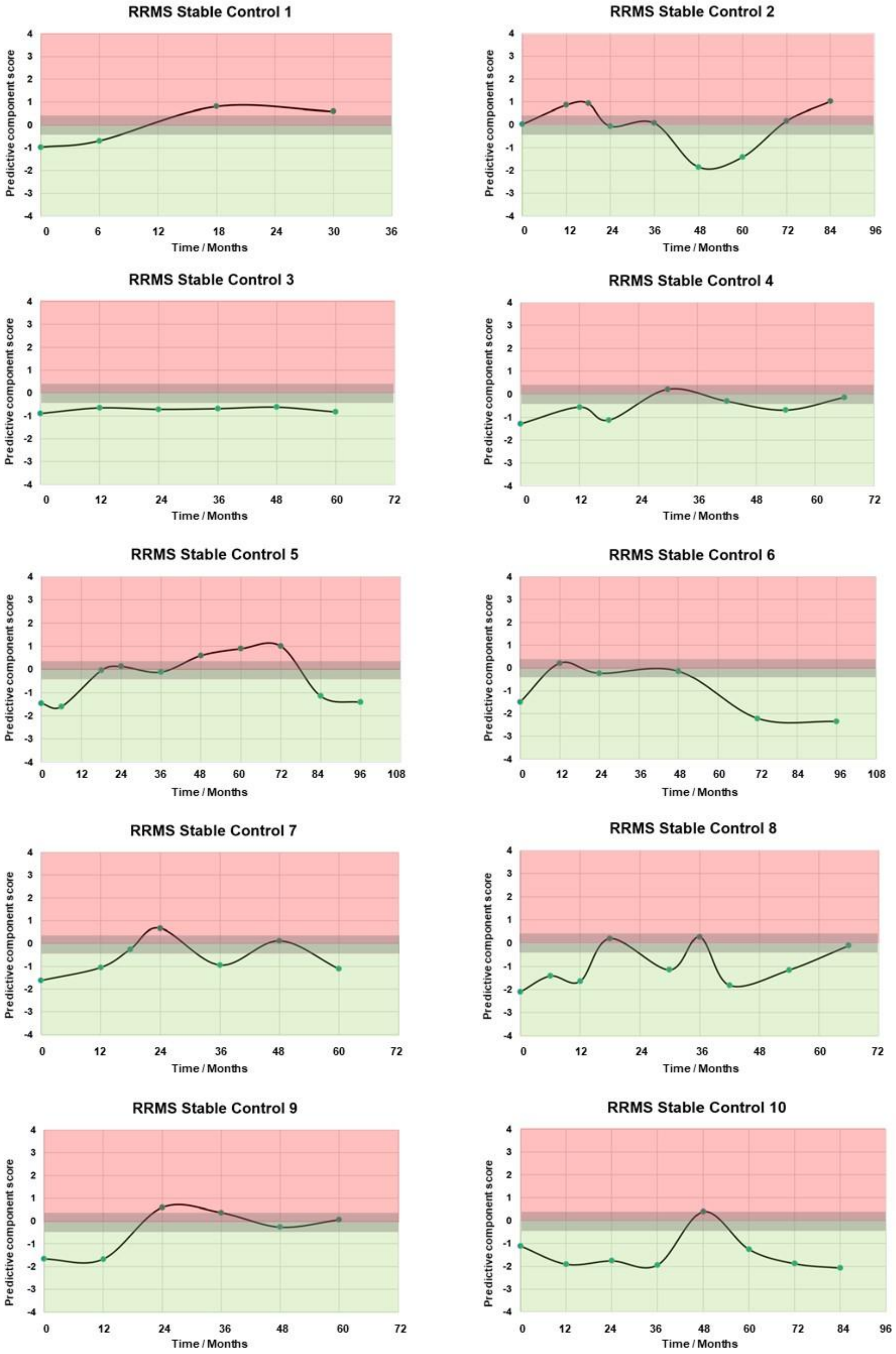


**Supplementary Fig. 2. Serum metabolomics as an alternative approach to stage MS.** Predicted OPLS-DA scores of 10 control individuals (stable RRMS) that were not used to construct the reference multivariate model. The red-shaded area represents the SPMS-like metabolome, while the green area corresponds to the RRMS-like metabolome (grey-shaded area represents the 95% CI). Each datapoint corresponds to a specific time point when the blood sample was collected.

### *Metabolomics facilitates the staging of multiple sclerosis in an individual*

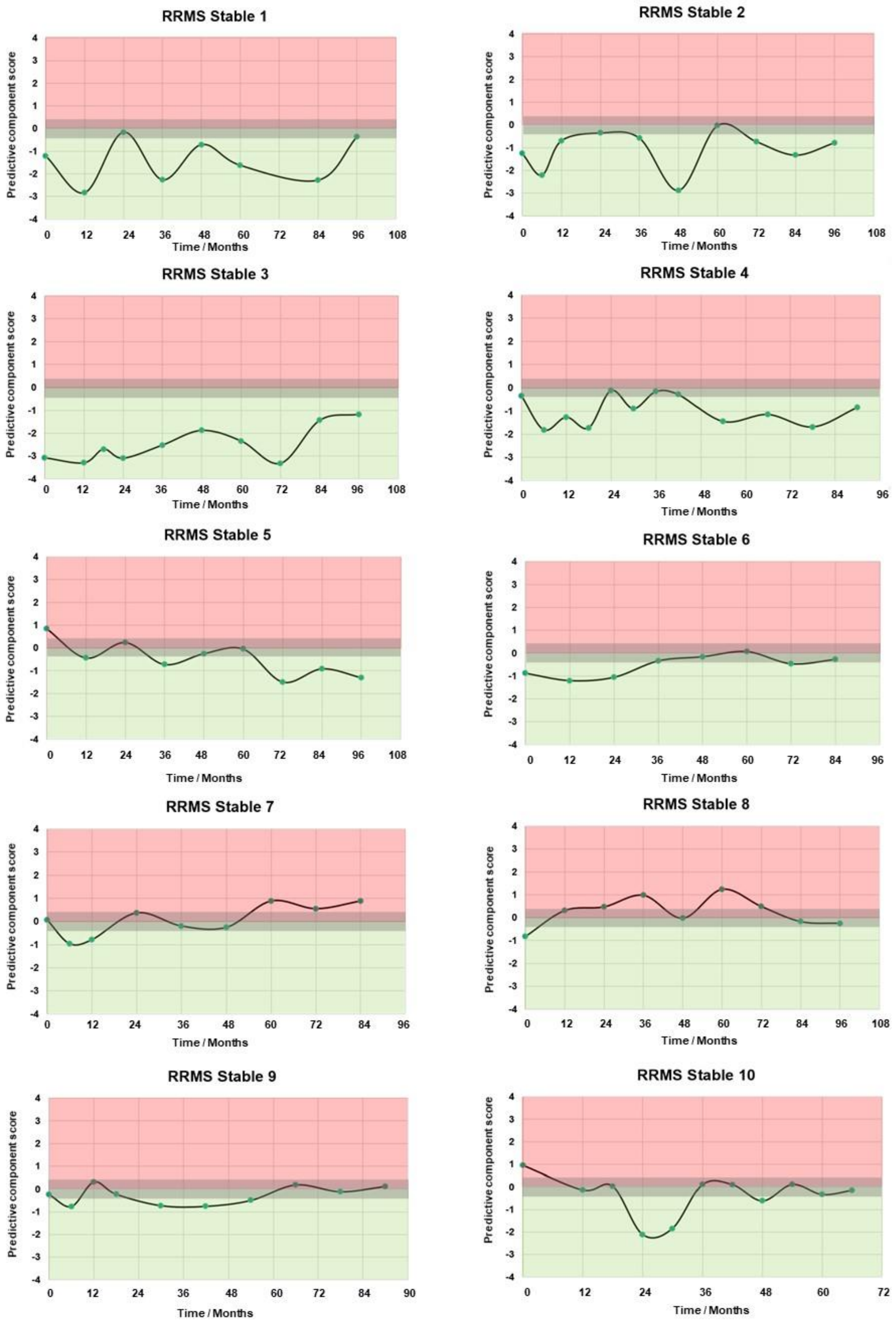


**Supplementary Fig. 3** Profile of ten stable RRMS (non-progressing) individuals, originally utilised to construct the reference multivariate model. Subsequently, each of these individuals was excluded from the original model, and a new diagnostic model was constructed to predict the position of the excluded individual. The red-shaded area represents the SPMS-like metabolome, while the green area corresponds to the RRMS-like metabolome (grey-shaded area represents the 95% CI). Each data point corresponds to a specific time point when the blood sample was collected. The predictive component score of each point represents the metabolomics profile at a given time point and was then plotted.

## *Metabolomics facilitates the staging of multiple sclerosis in an individual*

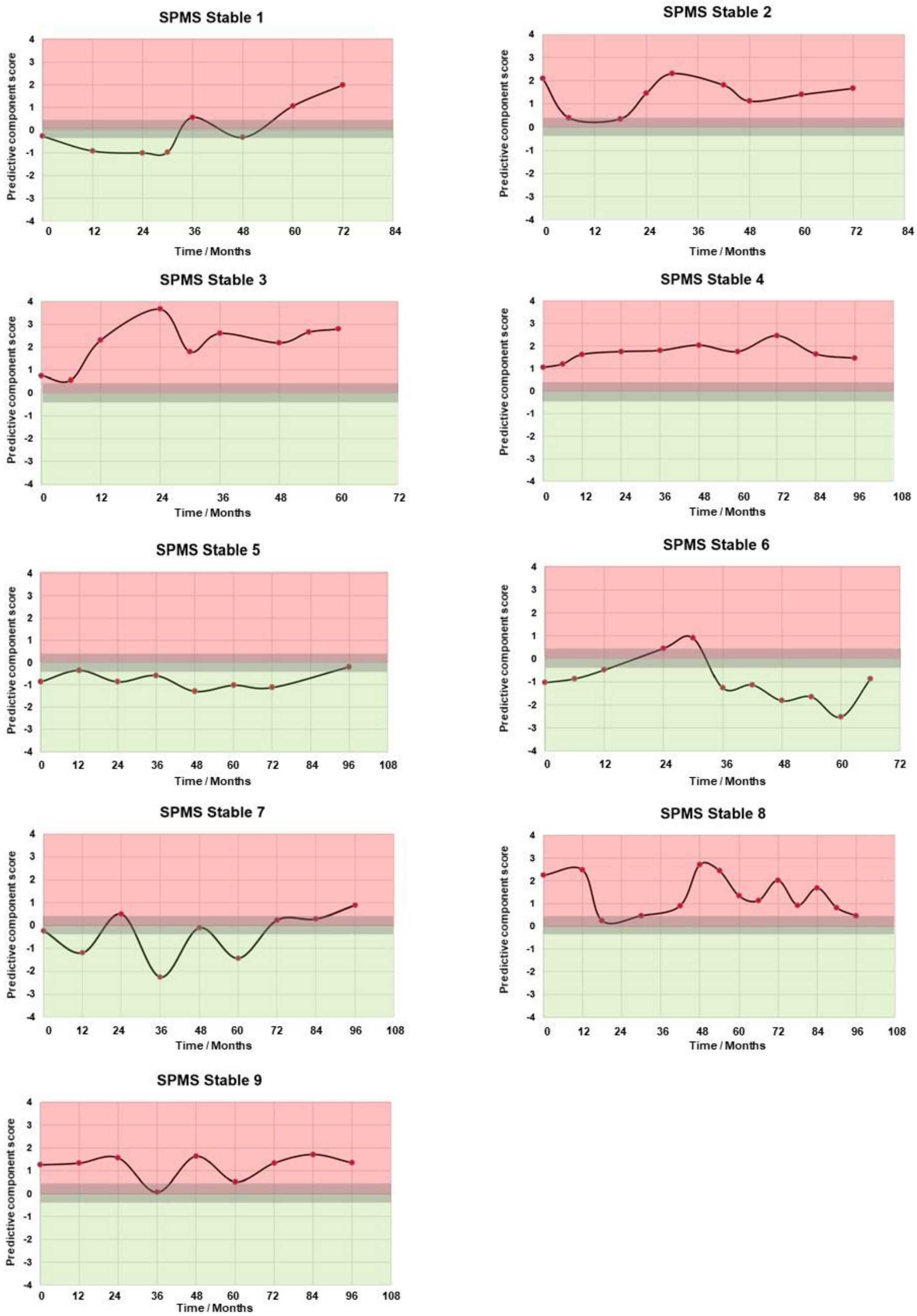


**Supplementary Fig. 4** Profile of ten non-transitioning SPMS (slow progressing) individuals, originally utilised to construct the reference multivariate model. Subsequently, each of these individuals was excluded from the original model, and a new diagnostic model was constructed to predict the position of the excluded individual. The red-shaded area represents the SPMS-like metabolome, while the green area corresponds to the RRMS-like metabolome (grey-shaded area corresponds to the 95% CI). Each data point corresponds to a specific time point when the blood sample was collected. The predictive component score of each point represents the metabolomics profile at a given time point and was then plotted.

### *Metabolomics integrated with sGFAP allows MS staging in an individual*

An OPLS-DA model discriminating between "pure" RRMS and SPMS individuals, involving sGFAP serum concentrations as an additional variable, was constructed. The *sub*-cohort consisted of 10 participants with stable RRMS and 9 participants with SPMS – for patient demographics, refer to Supplementary Table 2. Note: multiple samples from each individual (collected at different time points over an 8-year period) were used to construct this multivariate model to ensure adequate statistical power.

The integration of NMR metabolomics with serum concentrations of sGFAP augmented the discriminatory capacity of the multivariate model, thereby enhancing accuracy, sensitivity, and specificity parameters.

**OPLS-DA Model parameters:**

Model accuracy: 78.6 ± 1.8%

Model sensitivity: 79.5 ± 3.1%

Model specificity: 77.1 ± 2.4%

AUC (multivariable ROC – metabolites): 0.912 (95% CI 0.868-0.955)

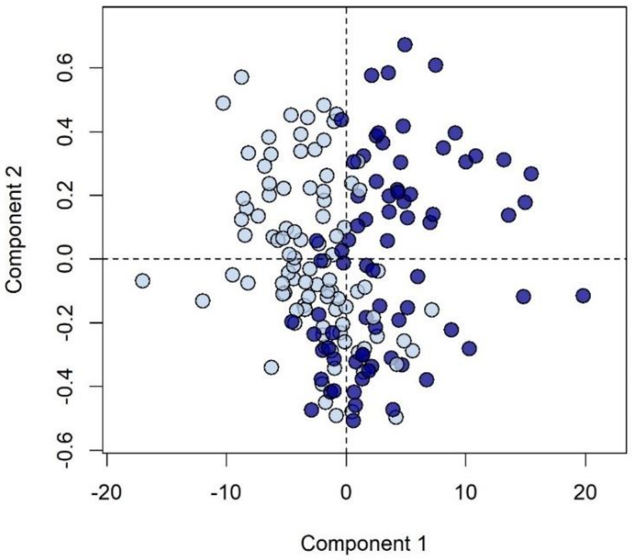


**Supplementary Fig. 5** OPLS-DA model discriminating between RRMS (dark blue) and SPMS (light blue) individuals (sGFAP was included as an additional variable in the original NMR metabolomics-based model)

**Random Forest Model parameters:**

Model accuracy: 82.1 ± 2.0%

Model sensitivity: 85.1 ± 2.6%

Model specificity: 81.2 ± 3.4%

***The baseline concentrations of five serum metabolites and sGFAP and sNfL predict the likelihood of MS disease stage change (transition from RRMS to SPMS)***

The Kaplan-Meier analysis revealed that baseline serum metabolites, along with sGFAP and sNfL concentration levels, have predictive capabilities for an individual's transition from RRMS to SPMS.

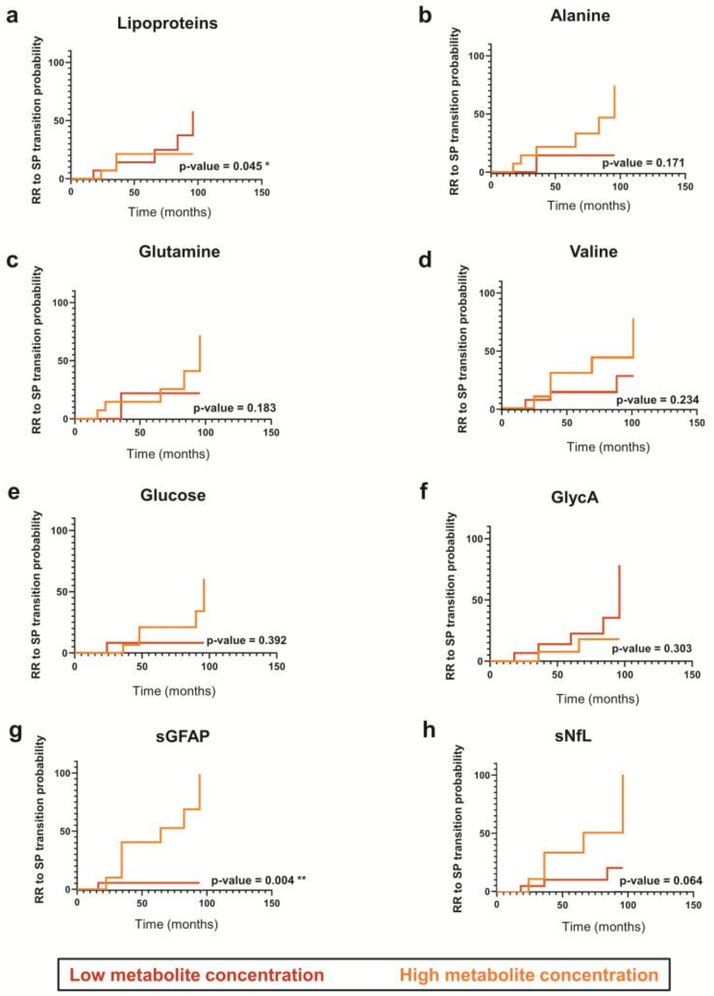


**Supplementary Fig. 6** Kaplan-Meier curves for the most discriminatory serum metabolites, sGFAP, and sNfL. The baseline concentrations of potential biomarkers were utilised, and the transition from RRMS to SPMS was defined as the event of interest, as depicted in the Kaplan-Meier curves. High baseline serum levels of the specified metabolite (using *z*-scores of each metabolite concentration) are depicted in yellow, while low baseline metabolite levels are represented in red.

### *Integration of sGFAP and NMR-metabolites enhances prediction of disease progression*

The predictive accuracy of the different biomarker combinations was assessed by repeating the Random Forest analysis using only the identified discriminatory variables. The mean AUC was calculated across all folds and repetitions to provide an average measure of model discrimination performance. To validate the significance of the model's AUC, a permutation test was performed where class labels were randomly shuffled to create a null distribution of AUC values. The model's AUC was compared against this distribution to confirm it performed better than random chance. The reported AUC represents the mean AUC obtained from the full dataset (all available samples; *i.e.,* not exclusively baseline samples) across all cross-validation runs. The reported ROC curves are the aggregate ROC curves generated by combining true labels and predicted probabilities from all cross-validation folds.

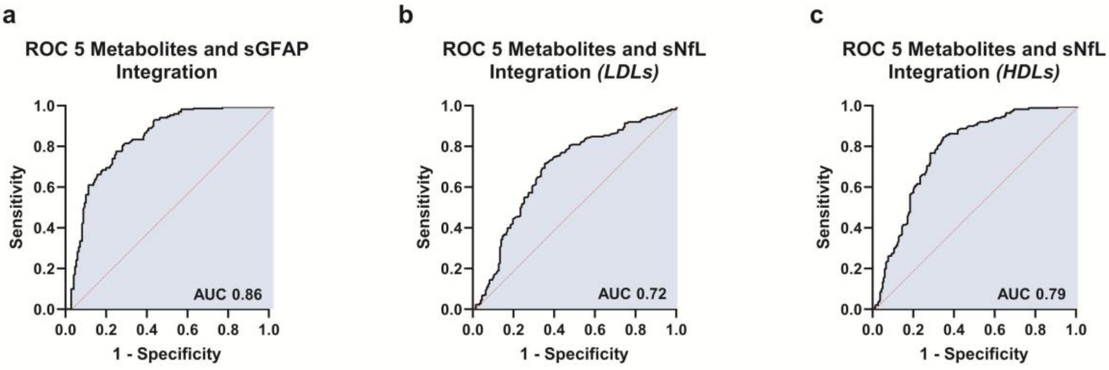


**Supplementary Fig 7. Improved classification of future progressors and non-progressors was achieved through the combination of five metabolites (LDLs, glutamine, valine, alanine, glucose) with sGFAP and sNfL.** (a) ROC curve for the integration of five serum metabolites (LDLs, alanine, glutamine, valine, glucose) and sGFAP. (b) ROC curve for the integration of five serum metabolites (***LDLs***, alanine, glutamine, valine, glucose) and sNfL.(c) ROC curve for the integration of five serum metabolites (***HDLs***, alanine, glutamine, valine, glucose) and sNfL.

**ROC curve Supplementary Fig. 7a LDLs, alanine, glutamine, valine, glucose and sGFAP**

AUC: 0.86 (95% CI 0.80 - 0.90)
Accuracy: 76.2 ± 1.1%
Sensitivity: 74.9 ± 1.6%
Specificity: 77.8 ± 1.5%

**ROC curve Supplementary Fig. 7b LDLs, alanine, glutamine, valine, glucose and sNfL**

AUC: 0.72 (95% CI 0.65 - 0.79)
Accuracy: 66.6 ± 1.4%
Sensitivity: 68.0 ± 1.8%
Specificity: 65.8 ± 2.4%

**ROC curve Supplementary Fig. 7c HDLs, alanine, glutamine, valine, glucose and sNfL**

AUC: 0.79 (95% CI 0.74 - 0.84)
Accuracy: 71.9 ± 1.5%
Sensitivity: 72.1 ± 1.8%
Specificity: 72.2 ± 2.3%

Interestingly, replacing LDLs with HDLs increased the ROC AUC to 0.79; however, this effect was not observed at baseline. This improvement might be attributed to collinearity between NfL and LDLs, with HDLs potentially capturing complementary information that the other variables do not provide.

## *Independent cohort validation confirms the metabolomic findings*

***Supplementary Table 6*** *Demographic characteristics of progressing and non-progressing MS individuals for the validation cohort (Oxford MET cohort). Non-progressors exhibited stable ambulatory function, maintaining unrestricted walking ability at both baseline and 3-year follow-up, whereas progressors experienced pronounced ambulatory decline, defined by a reduction in walking capacity to ≤500 meters. The individual characteristics, presented as mean ± standard error of the mean (SEM), were compared using Fisher's exact test for categorical variables and the Wilcoxon test for continuous variables that were not normally distributed. Statistical significance was determined with the following thresholds: *p < 0.05, **p < 0.01, ***p < 0.001.*

| **Parameter** | **Progressors** | **Non-progressors** | ***p*-value** |
|---|---|---|---|
| *N* | 19 | 24 | |
| Female (%) | 13 (68.4%) | 13 (54.2%) | 0.37 |
| Age first sample | 46.6 ± 10.8 | 43.4 ± 8.9 | 0.30 |
| BMI (baseline) | 25.8 ± 5.0 | 25.6 ± 4.9 | 0.92 |
| EDSS first sample | 3.5 ± 1.1 | 2.9 ± 1.1 | 0.11 |
| **Disease course** | | | |
| RRMS | 14 (73.7%) | 23 (95.8%) | |
| SPMS | 3 (15.8%) | 0 (0.0%) | 0.13 |
| PPMS | 1 (5.3%) | 0 (0.0%) | |
| CIS | 1 (5.3%) | 1 (4.2%) | |
| **Therapy - baseline** | | | |
| **Untreated** | **7** | **9** | |
| **Orals** | **7** | **8** | |
| | dimethyl fumarate (5) | dimethyl fumarate (7) | |
| | fingolimod (1) | fingolimod (1) | |
| | teriflunomide (1) | - | |
| **Monoclonal antibodies** | **3** | **1** | |
| | natalizumab (3) | natalizumab (1) | |
| **Platform therapies** | **2** | **6** | |
| | interferon-beta-1a (1) | interferon-beta-1a (1) | |
| | glatiramer acetate (1) | glatiramer acetate (5) | |
