# Supplementary material for: Serum GFAP and NfL augment a metabolomics-driven strategy for long-term prediction of multiple sclerosis progression
Source: Commun Med (Lond). 2026 Feb 25;6:182. doi: 10.1038/s43856-026-01453-5 (PMC13046805; doi:10.1038/s43856-026-01453-5)
Supplement: Supplementary file 3 — Description of Additional Supplementary files [file 43856_2026_1453_MOESM3_ESM.docx]

**Description of Additional Supplementary Files**

Supplementary Data 1: Source data underlying the main figures
